# A rare cause of intestinal obstruction: ileosigmoid knot

**DOI:** 10.11604/pamj.2014.19.21.3902

**Published:** 2014-09-09

**Authors:** Issam Yazough, Houssam Benhammane, Oussaid Morad, Pierlesky Elion Ossibi, Imane Toughrai, Said Ait Laalim, Ibn Majdoub Hassani, Khalid Mazaz

**Affiliations:** 1 Department of Surgery, Faculty of Medecine and Pharmacy in Fez, Univerity Sidi Mohammed Ben Abdellah, CHU Hassan II, Fez, Morocco

**Keywords:** Intestinal obstruction, ileosigmoid knot, sigmoid colon

## Abstract

Intestinal obstruction is common surgical emergency; ileosigmoid knot is a rare cause of intestinal obstruction (a loop ileum and sigmoid colon twisted around each other in a knot). We recorded a three cases in HASSAN II hospital in the last 3 years.

## Introduction

Double sigmoid volvulus ileocecal is a “node” created by a volvulus two intestinal segments. Sigmoid colon and ileum. It is a particular cause of acute intestinal obstruction associated with a high mortality requiring surgical management adapted and fast.

## Patient and observation

Our study focuses on three cases of occlusion on ileo-sigmoid volvulus collected twice for surgery B CHU Hassan II of Fez.

### Case 1

**Figure 1:**
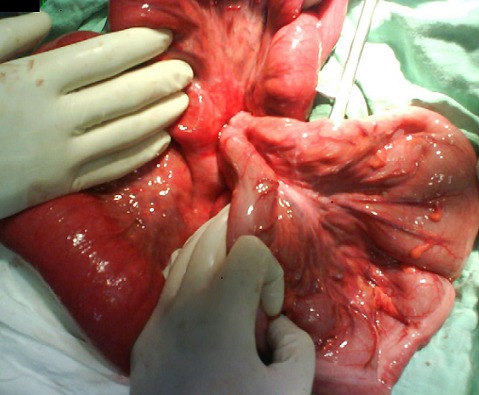
Views intraoperative node ileal around the base of the sigmoid

Patient 37 years, admitted to the emergency occlusive syndrome with the clinical examination and a significant asymmetric abdominal distension. The diagnosis of a double-colon volvulus Grelo was asked to scan before the combination of a sigmoid volvulus and a small bowel obstruction and lack of expansion of the sigmoid colon upstream. Colo-exsufflation first was performed. Surgical exploration showed a constriction of the last ileal loop the sigmoid to 3 cm from the ileocecal junction without digestive colon necrosis, followed by emergency laparotomy which confirmed the diagnosis double volvulus. The sigmoid colon caused necking of the last loop of the small intestine to 3 cm from the ileocecal junction (Figure 1). There was no intestinal necrosis. After dévolvulation, we performed a sigmoidectomy with colorectal anastomosis to prevent recurrence of the volvulus.

### Case 2

**Figure 2:**
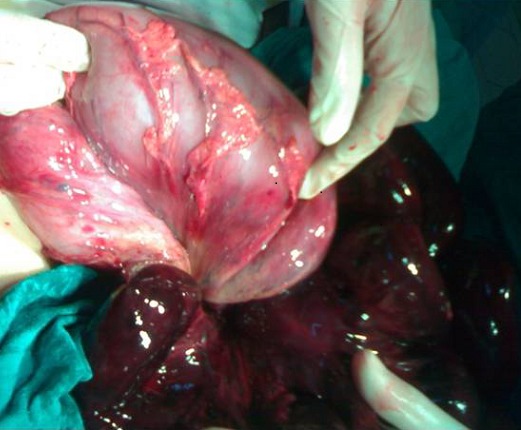
Other views intraoperative node ileal around the base of the sigmoid

65 year old patient admitted to the emergency room for an occlusive syndrome with shock. Abdominal examination revealed mild abdominal distension with generalized abdominal defense. After initial resuscitation ASP was performed and showed the presence of air-fluid levels such small bowel. Given the serious condition of the patient, CT was not performed. Diagnosis double sigmoid volvulus ileocecal was asked in the intraoperative discovery of ileo-sigmoid node. Sigmoid was not necrotic. Three meters from the distal ileum were necrotic and were resected with the cecum (Figure 2). End-to-side ileo colic was performed because of the brevity of the small intestine. We planned elective sigmoidectomy at a later date.

### Case 3

50 year old patient admitted to the emergency for acute abdomen. In the abdominal examination revealed generalized abdominal guarding with leukocytosis and elevated CRP, an ultrasound showed an outpouring of great abundance finely echo gene where the admission of the patient to the operating room with surgical exploration has objectified the presence of a double volvulus with necrosis ileosigmoideine 80 cm small bowel necrosis and sigmoid the patient underwent resection of a dévolvulation with hail and sigmoid confection with two stomas has Volkman boils right and left.

## Discussion

Double volvulus or volvulus ileo-sigmoid or ileo-sigmoid node in Anglo-Saxon (ileosigmoid knotting) literature, is a “node” created by a volvulus two intestinal segments sigmoid colon and small intestine, particularly the ileum. Ileosigmoid knotting (ISK) is a rare cause of intestinal obstruction that rapidly progresses to gangrene of the ileum as well as the sigmoid colon [1]. Three factors are responsible for the ISK: a long small bowel mesentery and freely mobile small bowel; a long sigmoid colon on a narrow pedicle; and finally the ingestion of high bulk diet in the presence of an empty small bowe [2, 3]. Rapid repletion of the jejunum promote its descent into the pelvis and twisting around the empty ileum and taking the sigmoid loop; peristalsis aggravate the node [2, 4]. Alver et al [5] classified the sigmoid volvulus ileo into four types: Type I is the most common and occurs when the ileum (asset) is volvule goshawks sigmoid colon. Type II sigmoid is volvule goshawks ileum. Type III occurs when the terminal ileum and cecum is volvule goshawks sigmoid. Double volvulus is unknown when we can not distinguish the asset liability among the different digestive segments. Types I and II are divided into subgroups A and B respectively distinguish volvulus clockwise anti-clockwise volvulus. The reported mortality from ISK varies from 0% to 48%. The mortality figures are generally related to the duration of symptoms, the presence or absence of gangrene and general status of the patient, including the presence of septicemic shock [2, 6].

## Conclusion

The ileo-sigmoid node is rarely a cause of acute intestinal obstruction, severe. Preoperative diagnosis by CT is not always obvious. Emergency surgery is needed.
